# Supplementary material for: Complexome profiling of the Chlamydomonas psb28 mutant reveals TEF5 as an early PSII assembly factor
Source: Plant Cell. 2025 Mar 17;37(6):koaf055. doi: 10.1093/plcell/koaf055 (PMC12164590; doi:10.1093/plcell/koaf055)
Supplement: koaf055_Supplementary_Data [file koaf055_supplementary_data.zip › psb28_interactive.zip!/plots/intensity/qualityPlots/peptides/_Cre12.g542350.t1.2.html]

### Cre12.g542350.t1.2

Trivial name:   
SeqCoverage [median]: 21.90  
Mass: 17.95 kDa  
  
 

| Sequence | MissedCleavage | Mass [Da] | wtIntensity | muIntensity | Score |
| --- | --- | --- | --- | --- | --- |
| AMDVPYQVELVR | 0 | 1418.72 | 0.00 | 4310.85 | 104.52 |
| LKAMDVPYQVELVR | 1 | 1659.90 | 872.46 | 1314.36 | 59.61 |
| MEGLPKR | 1 | 829.45 | 949.56 | 1129.53 | 66.99 |
| RHVLISVDDSPASMK | 1 | 1653.85 | 0.00 | 444.25 | 71.08 |
|  |  |  | 1822.0 | 7199.0 |  |
